# Early Failures Benefit Subsequent Task Performance

**DOI:** 10.1038/srep21293

**Published:** 2016-02-17

**Authors:** Hideyoshi Igata, Takuya Sasaki, Yuji Ikegaya

**Affiliations:** 1 Graduate School of Pharmaceutical Sciences, University of Tokyo, Tokyo, Japan; 2 Center for Information and Neural Networks, Suita City, Osaka, Japan

## Abstract

Animals navigate using cognitive maps. However, how they adaptively exploit these maps in changing environments is not fully understood. In this study, we investigated the problem-solving behaviors of mice in a complicated maze in which multiple routes with different intersections were available (Test 1). Although all mice eventually settled on the shortest route, mice that initially exhibited more trial-and-error exploration solved the maze more rapidly. We then introduced one or two barriers that obstructed learned routes such that mice had to establish novel roundabout detours (Tests 2/3). Solutions varied among mice but were predictable based on individual early trial-and-error patterns observed in Test 1: mice that had initially explored more extensively found better solutions. Finally, when the barriers were removed (Test 4), all mice reverted to the best solution after active exploration. Thus, early active exploration helps mice to develop optimal strategies.

Choosing an optimal strategy from many alternatives is a common requirement of life. As natural environments are continuously changing, animals need to adaptively make flexible decisions with regard to their current situations by updating their information and behavioral strategies on a moment-by-moment basis.

In spatial maze tasks, rodents can learn an efficient pathway toward a food reward. This goal-directed behavior depends on a cognitive process that internally represents the spatial information of the external world, known as a “cognitive map”^1^,^2^. The cognitive map is thought to develop via the accumulation of memories regarding action-to-outcome relationships and helps the animals navigate to produce a desired outcome. The cognitive process also enables prospective planning for *de novo* trajectories when animals are exposed to suddenly altered road networks through the emergence of new obstacles and shortcut pathways^3^,^4^,^5^,^6^.

Most early behavioral studies addressing spatial cognition used well-trained animals and simple spatial mazes with a few choice options, probably to reduce the complexity of data analysis and behavioral variability across individuals. In addition, these studies evaluated the behavioral characteristics simply by quantifying motor activity, such as moving distance, stay time, and angular velocity. However, true spatial navigation must involve more complicated problem-solving strategies because the natural world consists of multiple choice options with various outcomes and mental costs that may be continuously changing. Empirical evidence to support the cognitive map theory in such complex environments is required. To this end, efficient analytical strategies are necessary to identify significant factors that affect a variety of behavioral patterns.

In this study, we examined how mice learn to navigate from the start box to the reward goal in a spatial maze with asymmetrical choice points. For example, in the maze illustrated in Fig. 1a, it is easy for humans to identify that the most optimal pathway is Route #1 because we can view the entire maze structure from the top. However, are mice able to find the same shortest route through spontaneous explorations in the maze? If so, which form of learning behavior optimizes their strategies? Here, we report that mice spontaneously acquire the best route within tens of trials and that the number of early error trials was negatively correlated with the overall task performance. We then altered the learned route by adding one or two barriers so that the mice needed to find other new detour routes. Finally, we removed the barriers so that the mice had access to the previous optimized route. At each stage of test, we compared task performance and behavioral patterns, including trial-and-error exploration, erroneous routing, and vicarious trial-and error (VTE) behavior, which are likely associated with animals’ decision making and motivation for active sampling of task environments. Through a series of multivariate analyses, we identified the behavioral features that predicted subsequent task performance.

## Results

### Overview of behavioral tasks and analyses

**Figure 1:**
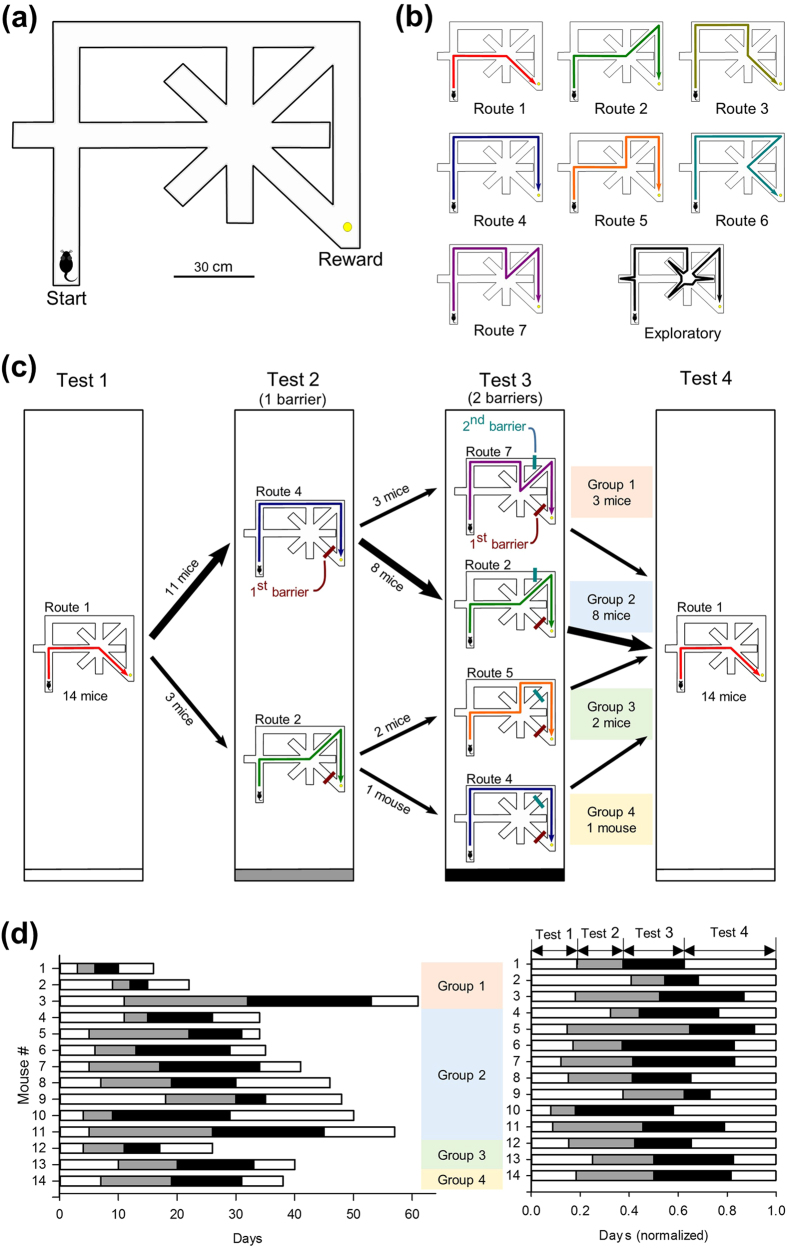
Description of the maze tasks used in this study. (**a**) Illustration of the maze. In each trial, an animal was first placed at the start position. A trial ended when the animal reached the goal position and took the reward. (**b**) Seven unicursal routes from the start to the goal without entering the same nodes more than twice. The route numbers are ordered according to the travel distance. Route 1 corresponds to the optimal (shortest) route. (**c**) A schematic diagram showing how 14 animals showed the most preferred routes at the end of the individual tests. In Test 2 and Test 3, the mice were exposed to a detour problem by the insertion of a barrier (1^st^ and 2^nd^ barriers). Animals were classified into four groups based on their most preferred routes. (**d**) Days required to complete the sequential four tests for the 14 mice. The mouse numbers were sorted by mouse groups. The right panel shows the same data normalized by the total number of days.

**Figure 2:**
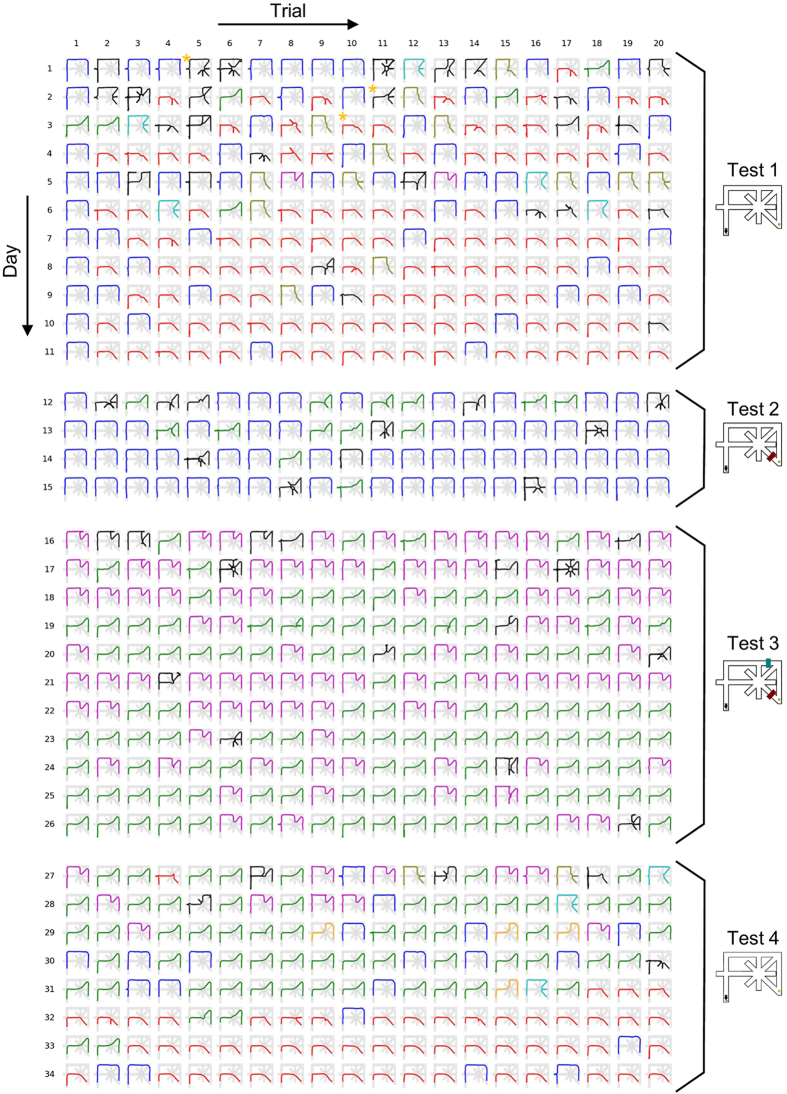
All of the trajectories observed from one representative animal (mouse #4). Thick colored lines represent the animal’s trajectories superimposed on the maze structure shown in gray. Days are ordered from top to bottom, and the twenty trials per day are ordered from left to right. The example trials used in Fig. 3c are marked by orange asterisks.

**Figure 3:**
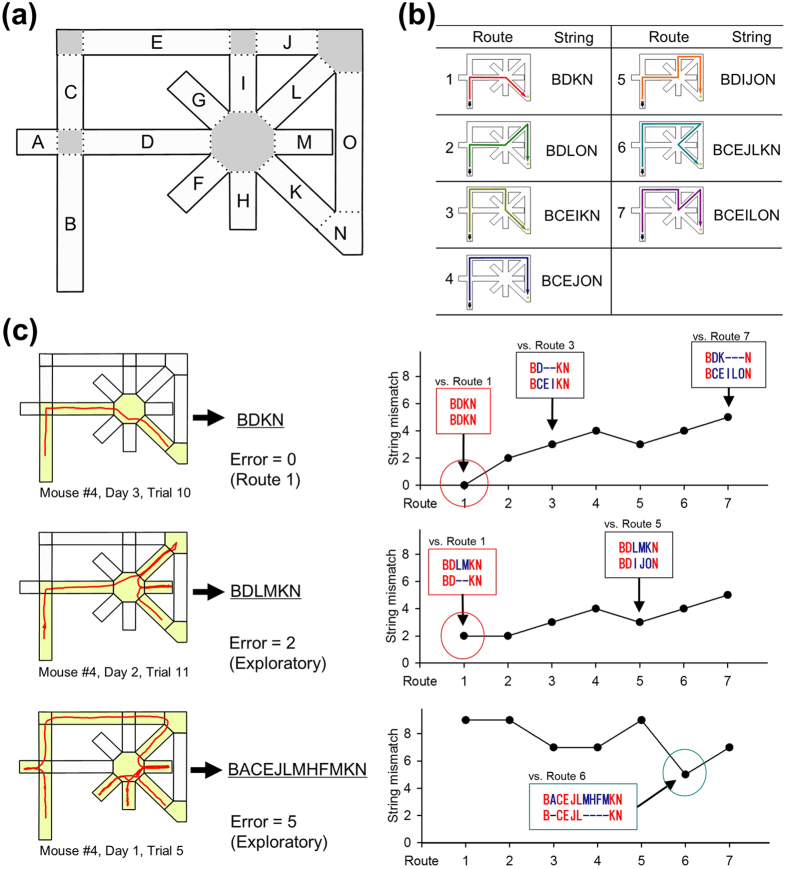
Analysis of animal’s trajectory. (**a**) The maze was divided into 15 segments that are labeled with characters A–O. The hinge segments were not labeled and are shown in gray. (**b**) Each unicursal route shown in Fig. 1b was converted into a character string (template string). (**c**) Three examples showing how animals’ trajectories were analyzed. A trajectory in each trial was first converted into a character string (target string, left), and the numbers of mismatches were counted between the target string and individual template strings (right). If the lowest number of mismatches was less than 2, the animal’s trajectory was considered to be the route with the least mismatch. If the lowest number of mismatches was 2 or over, the trajectory was considered to be an exploratory route.

**Figure 4:**
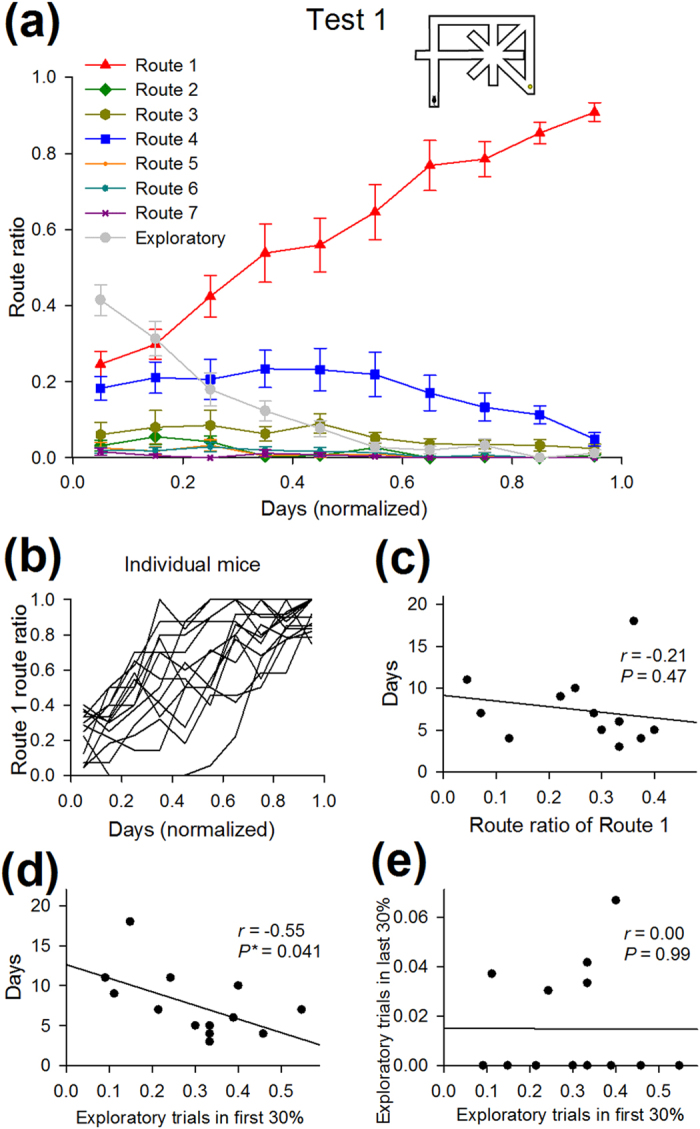
Learning time course and behavioral characteristics in Test 1. (**a**) Average percentage of choosing individual routes (route ratios) plotted against the normalized testing days (n = 14 mice). Data are presented as the mean ± the standard error of the mean. (**b**) Time changes in the route ratio of Route 1 for individual mice. Each line represents a mouse. (**c**) No correlation was found between the route ratio of Route 1 on the first day and the number of days to reach the learning criterion. Each dot represents a mouse. (**d**) Days to reach the learning criterion were correlated with the number of exploratory trials in the first 30% period. (**e**) There was no correlation between the number of exploratory trials in the first 30% and last 30% periods.

The maze used in this work is shown in Fig. 1a. In initial habituation, mice were allowed to forage freely in the maze with food pellets placed on individual nodes for 10 min per day for 7–8 days. In subsequent behavioral tests, a reward was placed at the goal location for every trial. In Test 1, each trial started when a mouse was placed at the start position, and the trial ended when the animal reached the food reward. In a day, mice were tested in 20 trials. The maze was cleaned with ethanol after completing 20 trials once per day per animal, unless otherwise specified. In the maze task, there were seven different unicursal routes that the animal could take without re-visiting the same nodes (Fig. 1b). All 14 animals eventually took Route 1, the shortest route, in more than 75% of the trials (Fig. 1c). After completing Test 1, Test 2 was initiated with a barrier placed at the segment K (1^st^ Barrier). The most chosen detour route was defined as the most preferred route. Eventually, 11 and 3 animals showed their most preferred route as Route 4 and Route 2, respectively (Fig. 1c). After completing Test 2, Test 3 was performed with another barrier (2^nd^ Barrier). In Test 3, the most preferred detour routes varied depending on the animals and the barrier positions, including Routes 2, 4, 5 and 7. After completing Test 3, Test 4 was performed after the two barriers were removed. Thus, Test 4 was virtually identical to Test 1. In Test 4, all 14 animals eventually restored Route 1 as their most preferred route. Data of an example animal are shown in Fig. 2. To analyze the behavioral pattern, the trajectory in each trial was described as a character string, termed as a target string (Fig. 3). In each trial, we analyzed the numbers of mismatches between the target string and the template strings of the seven unicursal routes and defined the template route with the least mismatch as the closest route and the number of the least mismatch as the number of errors. If the number of errors was 1 or less, the animal’s trajectory was regarded as the closest route. If the number of errors was 2 or more, the trial was considered an exploratory (trial-and-error) trial. On each day, the probability of choosing a given route was defined as a route ratio (Fig. 4a).

### Initial habituation to the maze

In preliminary habituation periods, the mice were given ample opportunities to forage freely in the maze for 10 min per day for 7–8 days. On each habituation day, food pellets were provided on all nodes of the maze to facilitate animals’ motivation of exploration (Supplementary Fig. 1a). In these periods, animals nearly homogeneously covered all the 15 segments of the maze (Supplementary Fig. 1b). The average travel distance in a habituation day ranged 39.5 to 81.4 m, with an average of 59.7 m. The travel distance during habituation periods correlated with the average number of exploratory trials in the initial phase of Test 1 (Supplementary Fig. 1c), suggesting that animals that traveled more in habituation tended to show more exploration in subsequent tests. Neither the travel distance nor stay uniformity during habituation had significant effects on the entire behavioral performance in the subsequent test (Supplementary Fig. 1e–h).

### Learning the optimized route in Test 1

Behavioral data were obtained from 14 mice that were tested for a total of 548 days. After the habituation training, the mice were tested in 20 trials per day, from the start to the rewarded goal. In Test 1, the optimal strategy for solving the task was to take Route 1, the shortest route (Fig. 1b). As shown in an example in Fig. 2, the animals’ trajectories gradually became Route 1 over multiple days. Using template matching, which is generally used to determine DNA sequence homology, we classified a given trajectory into one of seven unicursal routes, as shown in Fig. 1b (for details, see Methods and Fig. 3). The term “unicursal route” refers to a route that did not allow revisiting the same location. A trajectory that included at least one revisiting pattern was defined as trial-and-error exploration.

The mean learning curve is shown in a time-evolution plot of the percentage of each route (a route ratio) as a function of days (Fig. 4a). For each mouse, the total days spent until reaching the criterion (i.e., until the ratio of one route exceeded 75% for 2 consecutive days) was normalized to 1. Then, the data were pooled from all 14 mice. All 14 mice tested learned to take Route 1. The learning period was 7.5 ± 1.1 days (ranging from 3 days to 18 days; *n* = 14 animals).

The learning curve indicates several characteristics. First, from the first few trials, most (10/14) animals chose Route 1, with a route ratio of more than 0.20 (Fig. 4b). Additionally, even on the first day, the trajectories rarely included backward movement. This initial non-random route might come from latent learning during habituation training^7^. No correlation was found between the route ratio of Route 1 on the first day and the days until reaching the learning criterion (Fig. 4c *r* = –0.21, *P* = 0.47), indicating that task performance on the initial day was not predictive of later behavioral performance. Second, in the early learning phase, the animals tended to show preferences for two routes; the first route was Route 1, and the second route was Route 4, the fourth shortest route (Fig. 4a). In contrast, other routes, such as Route 2 and Route 3, although they were shorter than Route 4, were rarely chosen. A possible explanation for this result is that Route 4 was the simplest for mice to solve the maze task because it did not require turns at eight-arm choice points. Choosing Route 4 may be an efficient strategy when animals are not yet confident regarding the task rules. Third, the animals showed a gradual, but not drastic, increase in the Route 1 route ratio (Fig. 4a). On average, it took 145 ± 21 trials until the mice reached the learning criterion from the trial that the mice first discovered Route 1. This trend suggests that the behavioral strategy did not immediately converge onto a single pattern. Even after the mice found an efficient route, they still appeared to attempt to seek the possible presence of a potentially better route.

Are there behavioral signs associated with the variability of the learning time course across animals? We found that the number of exploratory trials in the initial phase (during the first 30% of the trials) negatively correlated with the number of days until the animals reached the learning criterion (Fig. 4d; *r* = –0.55, *P* = 0.041). Consistent with this result, the number of errors per trial in the initial phase tended to correlate negatively with the number of days (Supplementary Fig. 2a; *r* = –0.51, *P* = 0.060), nor did motor activity in the initial phase, such as the total travel distance or the total angular velocity, have a significant correlation with later behavioral performance (Supplementary Fig. 2b,c). Taken together, these results highlight a functional significance of trial-and-error foraging in an early learning phase; that is, early erroneous explorations can benefit later task performance. No correlation was found between the number of exploratory trials in the initial and last phase (during the last 30% of the trials) (Fig. 4e; *r* = 0.00, *P* = 0.99).

**Figure 5:**
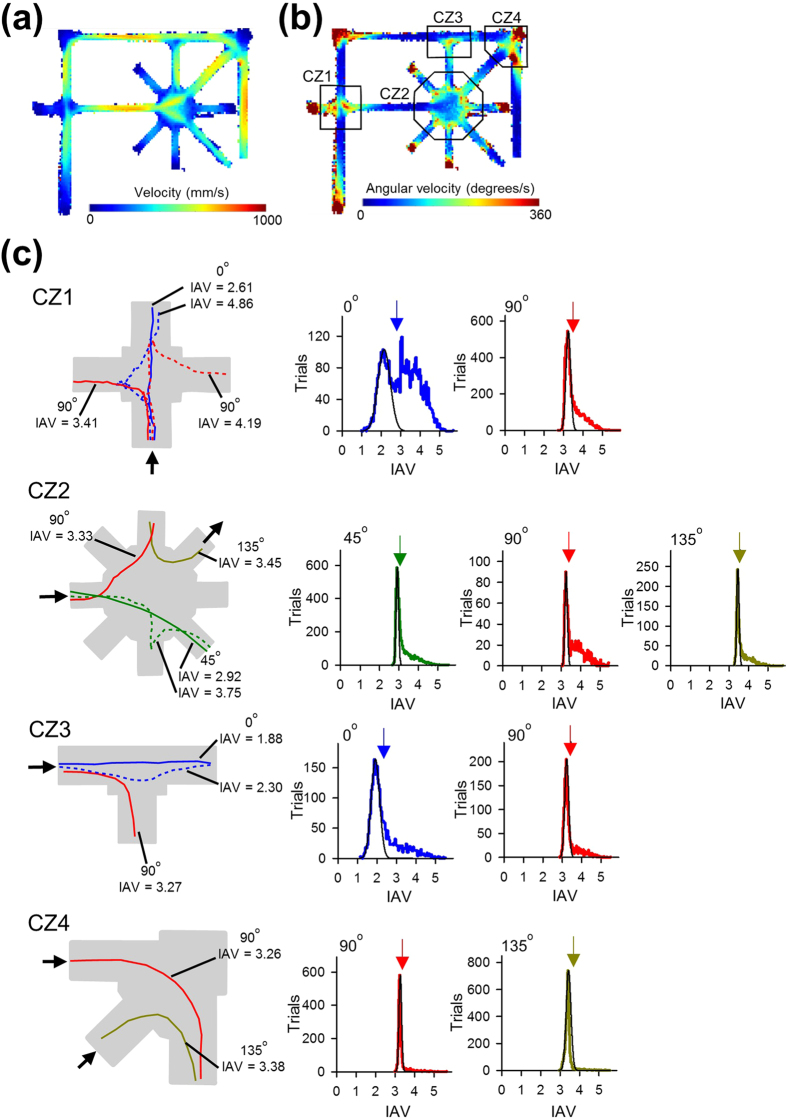
Detection of VTE events in choice zones. (**a,b**) Spatial maps showing the distribution of the velocity (**a**) and angular velocity (**b**) averaged across all animals. The color scale is from blue to red. Based on the angular velocity, VTE events were detected in four choice zones (CZ1-4). (c) (Left) Representative trajectories (lines) superimposed on each choice zone (gray). In each trajectory, log(*IdPhi*) is calculated and defined as an integrated angular velocity (IAV). Solid lines and dotted lines represent trajectories defined as non-VTE and VTE events, respectively. (Right) Distribution of IAV in each travel angle calculated from all animals. The black thin line represents a Gaussian curve by fitting to the distribution ranging from the lowest value to the mode value. The arrow on the top indicates the value at the 5% significance level to detect VTE events. The positively skewed distribution over the significance level was classified as VTE events.

**Figure 6:**
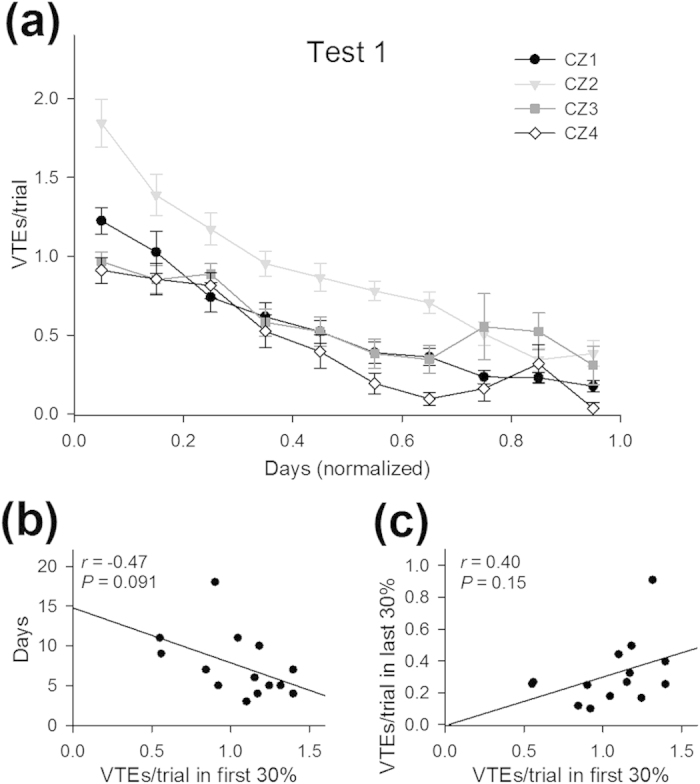
Analysis of the VTE events in Test 1. (**a**) The number of VTE events per trial at each choice zone plotted against normalized days. Data are presented as the mean ± the standard error of the mean. (**b,c**) Same as in Fig. 4 (**d,e**), but plotted for the number of VTE events per trial.

In addition to the apparent trial-and-error exploratory behaviors, animals are known to exhibit VTE behavior, in which they briefly pause at choice points and turn their head toward optional routes or, in some cases, even shortly enter into an alleyway but quickly change their choice. VTE has been suggested to reflect active sampling behaviors to scan a possible alternative pathway before reaching a decision^8^,^9^,^10^,^11^,^12^,^13^. We detected VTE events at four choice zones by measuring changes in the angular displacement of animals’ trajectories (for details, see Methods and Fig. 5). The VTE events at all of the choice zones were gradually reduced as the day went on, resulting in less VTE events in the last phase compared with the initial phase (*t*_13_ = 10.40, *P* = 1.1 × 10^−7^), suggesting a relationship between the number of VTE events and task performance (Fig. 6a). However, the number of VTE events in the initial phase tended to show a negative correlation with the days until reaching the learning criterion, but not significantly (Fig. 6b; *r* = –0.47, *P* = 0.091). No correlation was found between the number of VTE events in the initial and the last phases (Fig. 6c; *r* = 0.40, *P* = 0.15).

The results shown above were performed in the condition in which the maze was cleaned with 70% ethanol every 20 trials. Thus, mice might be guided by lingering sensory cues (e.g. scent cues) in the maze. We rule out this possibility based on the following three observations. First, on the last two days of Test 1, when the mice reached steady behavioral performance, the percentage of choosing Route 1 and the number of VTE events did not vary across trials within a day (Supplementary Fig. 3a; Route 1 route ratio, *F*(19,279) = 0.91, *P* = 0.57; VTE events, *F*(19,279) = 0.63, *P* = 0.88), suggesting that repeating the same tracks did not affect the subsequent route selection. Second, neither motor activity nor behavioral performance differed between two animal groups tested in the conditions in which the maze was cleaned every trial (*n* = 8 animals, ET group) and in the conditions in which the maze was cleaned every 20 trials (*n* = 14 animals, 20T group) (Supplementary Fig. 3b–f). Moreover, the two groups did not differ in the number of days until the animals reached the learning criterion also did not differ (Supplementary Fig. 3b; ET group, 6.8 ± 1.1 days, ranging from 4 days to 14 days; 20T group, 7.5 ± 1.1 days, ranging from 3 days to 18 days; t_20_ = 0.45, *P* = 0.66), the percentage of taking Route 1, Route 2, and Route 4 in the last two days (Supplementary Fig. 3c; Route 1, t_20_ = −0.23, *P* = 0.82; Route 2, t_20_ = −0.60, *P* = 0.55; Route 4, t_20_ = 0.92, *P* = 0.37), the percentage of taking the same route in two successive trials (Supplementary Fig. 3d; t_20_ = −0.39, *P* = 0.70), the choice variability at individual choice points (Supplementary Fig. 3f; CP1, t_20_ = 0.62, *P* = 0.54; CP2, t_20_ = −2.07, *P* = 0.052; CP3, t_20_ = −0.45, *P* = 0.65; CP4, t_20_ = −1.42, *P* = 0.16;), or the cumulative distribution of the time elapsed to complete a Route 1 trial in the last two days (Supplementary Fig. 3e; *D*_max_ = 0.098, *P* = 0.058, Kolmogorov-Smirnov test). Third, we performed additional experiments in which cleaning of the maze was performed every 2 trials and verified that Route 1 route ratio was not significantly different between trials after cleaning (cleaning trials) and trials after no cleaning (no cleaning trials) (Supplementary Fig. 3g; t_6_ = −1.37, *P* = 0.22). The fact that behavioral performance did not depend on the frequency of cleaning the maze suggests that mice are unlikely to exploit sensory cues on the maze to solve the task. Consistent with this idea, mice did not apparently exhibit sniffing behaviors during the task, and more than 80% of the trials in the last phase of Test 1 completed within 2.5 s (Supplementary Fig. 3e, typical cases shown in Supplementary Movie 1). The movie indicates that the mice run through the maze quickly at the nearly maximal speed without showing behavioral signs related to explorations of scent cues.

### Learning process to discover a solution for a detour problem

**Figure 7:**
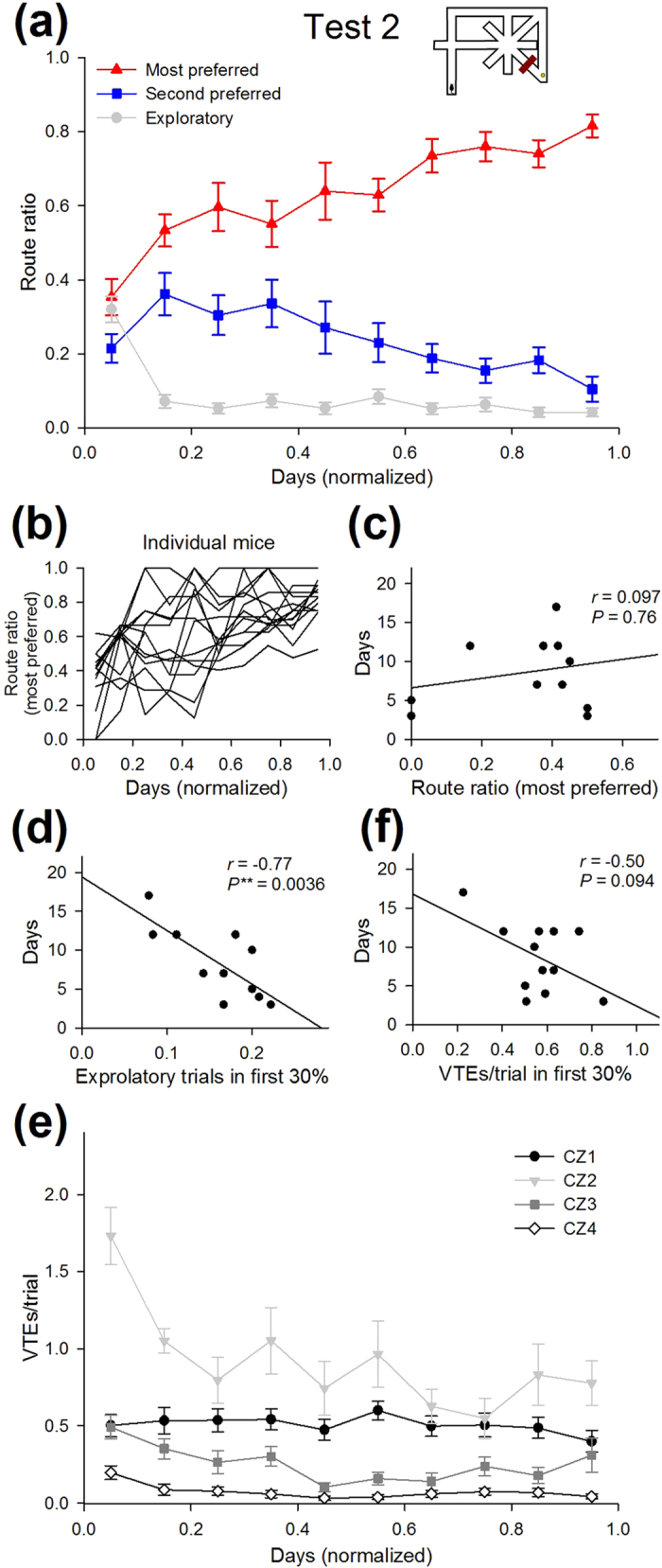
Learning time course and behavioral characteristics in Test 2. (**a**) The route ratios of the most preferred and second most preferred routes plotted against the normalized days in Test 2. The preferred routes were determined at the end of Test 2 and differed among mice. (**b–d**) Same as in Fig. 4b–d, but plotted for the most preferred route in Test 2. There was a significant negative correlation between the number of exploratory trials in the first 30% of the trials and testing days. In these plots, two animals were excluded as they did not meet the learning criteria within 21 testing days. (**e**,**f**) Same as in Fig. 6a,b, but plotted for datasets in Test 2. All data are presented as the mean ± the standard error of the mean.

After completing Test 1, we examined how the animals adapted their behaviors when the optimized route became unavailable (Test 2). We introduced an untransparent barrier in segment K such that the animals could not take Route 1 (Fig. 1c, 1^st^ barrier in Test 2) and needed to develop a new strategy for detouring to the rewarded goal. During repetitive exposures to the modified maze, 3 mice eventually switched their most preferred route to Route 2 (the second shortest route), whereas the remaining 11 mice took Route 4 (a less-choice-point, peripheral route) at the end of Test 2 (Figs 1c and 7a). Note again that Test 2 was completed when a mouse took the same route (either Route 2 or 4) in more than 75% of the trials for 2 consecutive days. To reach the learning criterion, animals required 10.4 ± 1.6 days (ranging from 3 days to 21 days; *n* = 14 animals). Similar to the case in Test 1, the route ratio of the route that was ultimately selected in Test 2 was already the highest among all of the detour routes from the first day of Test 2 and gradually increased to the level of the learning criterion (Fig. 7a,b). As in Test 1, the mice seemed to focus on two routes (the most preferred one and the second most preferred one) and rarely took the other routes from the beginning of Test 2. Animals may prefer choosing between two options because comparisons among more than two options may be emotionally taxing. No correlation was found between the route ratio of the most preferred route on the first day and the days to reach the learning criterion (Fig. 7c; *r* = 0.097, P = 0.76).

Consistent with the results of Test 1, the number of days spent to reach the learning criterion in Test 2 was negatively correlated with the number of exploratory trials in the initial phase of Test 2 (Fig. 7d; *r* = –0.77, P = 0.0036), which strengthens our idea that trial-and-error exploratory behaviors in an early learning stage contribute to shortening the learning period. The number of the total VTE events in the initial phase was significantly higher than that in the last phase (*t*_13_ = 2.9, P = 0.012). The number of VTE events at choice zone 2 (CZ2) was prominently greater than those in the other choice areas throughout Test 2 (Fig. 7e). This result is probably because the well-trained mice had first attempted to take Route 1 and encountered the barrier at segment K, which required experience to determine an alternative pathway at CZ2. Similar to Test 1, there was a weak but not significantly negative correlation between the number of VTE events in the initial phase in Test 2 and the days to reach the learning criterion (Fig. 7f; *r* = –0.50, P = 0.094).

**Figure 8:**
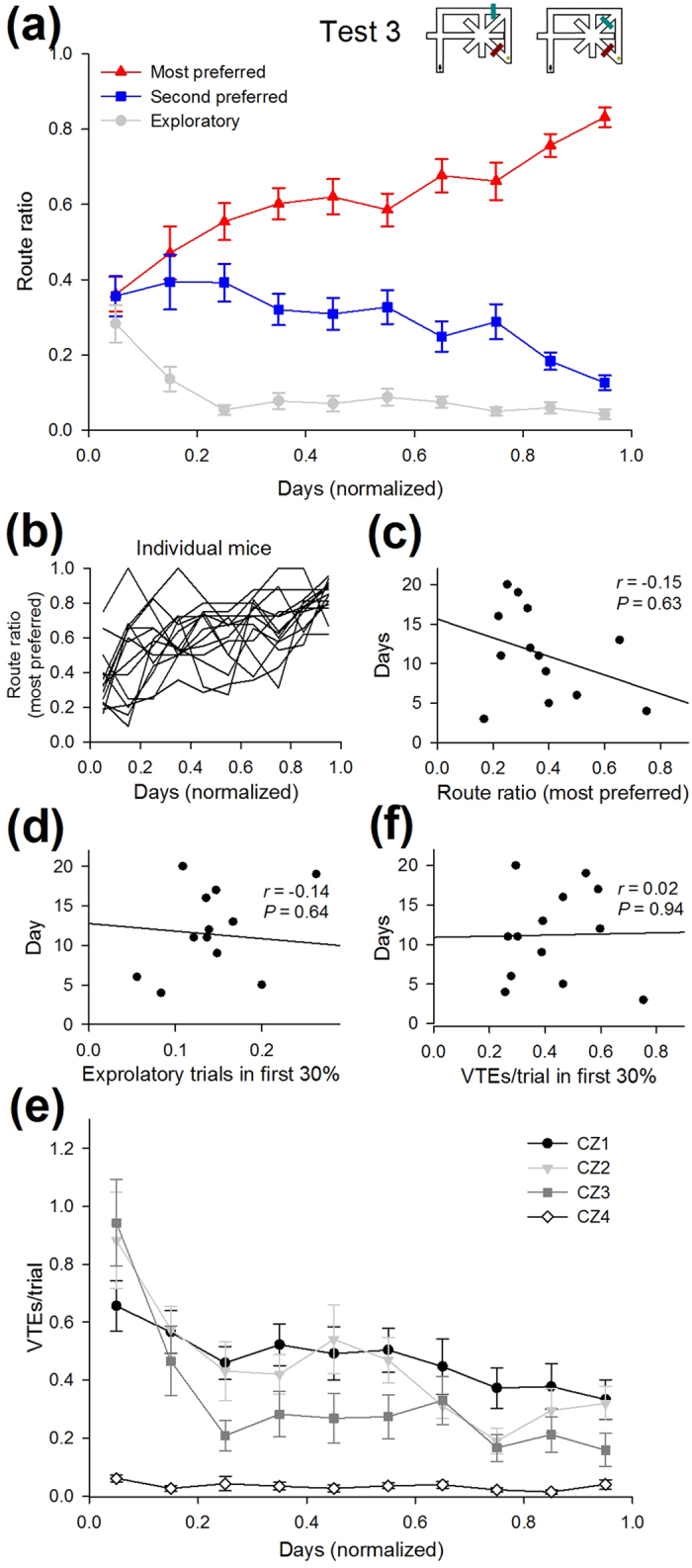
Learning time course and behavioral characteristics in Test 3. All of the figures are shown as in Fig. 7, but for the datasets in Test 3. No significant correlations were found in any of the plots. In these plots, one animal was excluded as it did not meet the learning criteria within 21 testing days. All data are presented as the mean ± the standard error of the mean.

After the completion of Test 2, an additional barrier was introduced at segment J or L to block the most preferred route taken in Test 2 (Fig. 1c, 2^nd^ barrier in Test 3). Specifically, every mouse was left with two detour routes, Route 2 and Route 7 (*n* = 11 mice) or Route 4 and Route 5 (*n* = 3 mice). The detour routes that individual animals eventually took are shown in Fig. 1c; 8 and 3 of 11 mice eventually switched their most preferred route from Route 4 to Route 2 and Route 7, respectively, whereas 2 and 1 of 3 mice changed from Route 2 to Route 5 and Route 4, respectively (Figs 1c and 8a). The learning time course in Test 3 appeared to be similar to that in Test 2, and the animals required 11.9 ± 1.6 days (ranging from 3 days to 21 days; *n* = 14 animals) to reach the learning criterion (Fig. 8a). The route ratio of the most preferred route defined in the last day of Test 3 was 0.36 ± 0.05 on the first day of Test 3 (Fig. 8b). There was no correlation between the route ratio on the first day and the number of testing days (Fig. 8c; *r* = –0.15, P = 0.63). Unlike Test 1 and Test 2, no correlation was found between the number of exploratory trials in the initial phase and the number of days in Test 3 (Fig. 8d; *r* = –0.14, P = 0.64). This finding is presumably because only two routes were available in Test 3 and required no more need of trial-and-error behaviors. The total number of VTE events in the last phase was significantly lower than that in the initial phase (Fig. 8e; *t*_13_ = 6.8, *P* = 1.3 × 10^−5^). No correlation was detected between the number of VTE events in the initial phase and the number of testing days in Test 3 (Fig. 8f; *r* = 0.02, *P* = 0.94).

### Behavioral process to restore the optimized route

**Figure 9:**
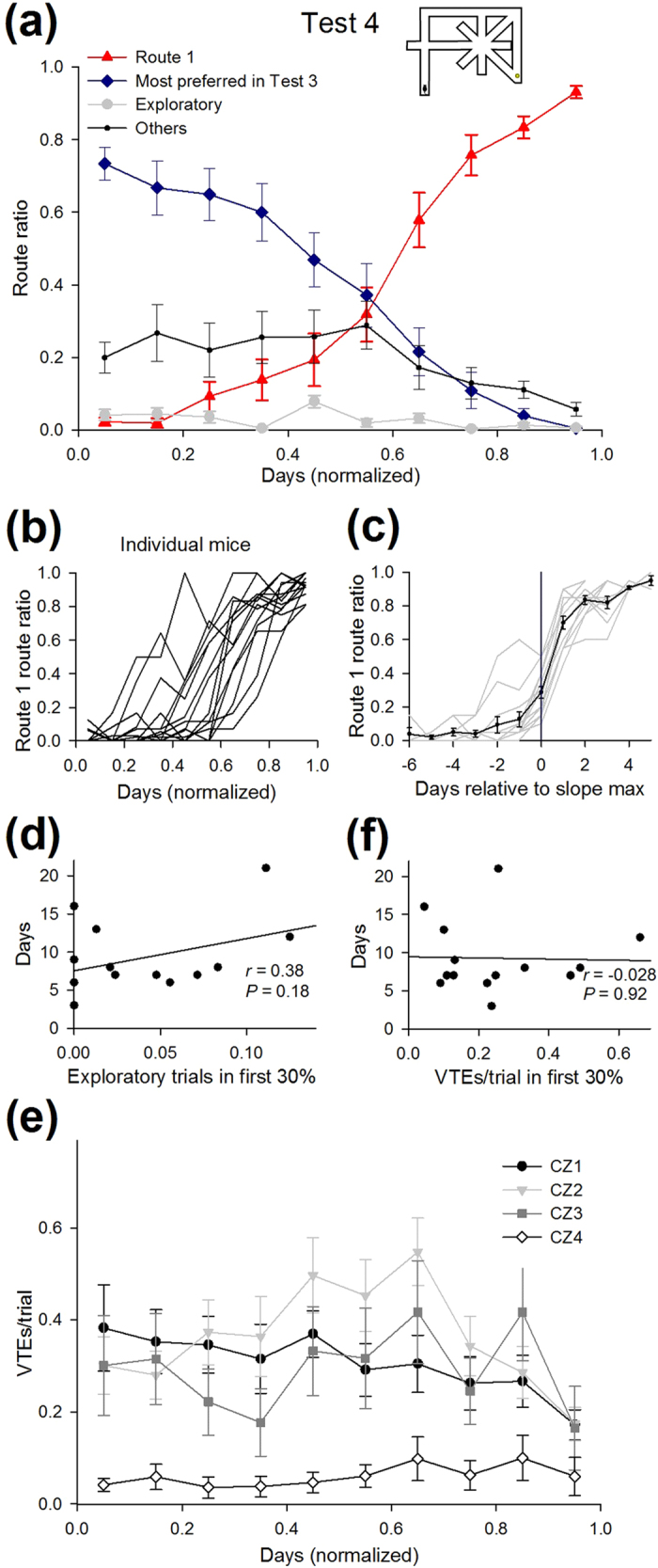
Learning time course and behavioral characteristics in Test 4. (**a**) Route ratios of Route 1 and the most preferred routes observed in Test 3. The other routes were classified as Others. (**b**) Same as in Fig. 4b, but plotted for Route 1 in Test 4. Each line represents a mouse. (**c**) The learning curve of Route 1 aligned to the day with the maximum slope of the curve in each animal. (**d**–**f**) Same as in Fig. 8d–f, but plotted for datasets in Test 4. No correlations were found in any of the plots. All data are presented as the mean ± the standard error of the mean.

**Figure 10:**
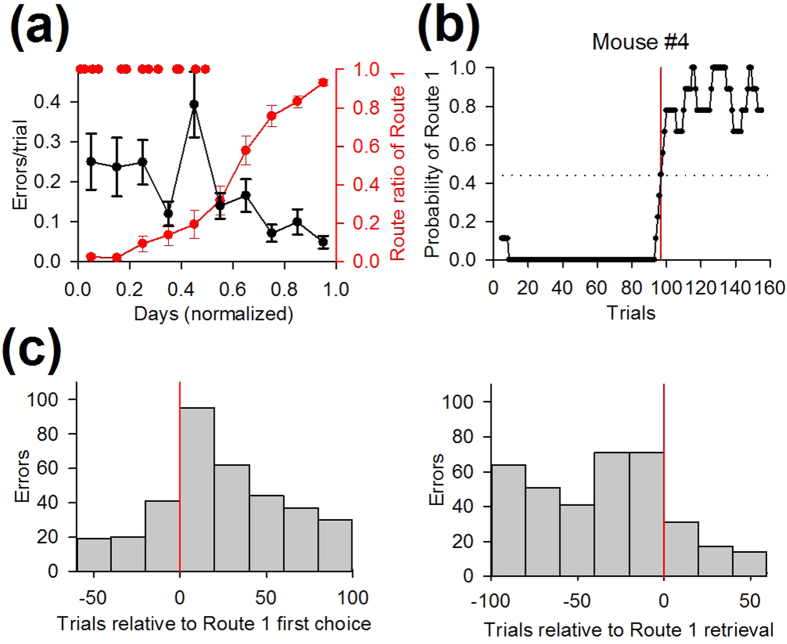
A transient increase in erroneous behavior was associated with the behavioral performance in Test 4. (**a**) The number of errors plotted against normalized days in Test 4. The learning curve of Route 1, which is similar to Fig. 9a, is superimposed on the graph. Red dots shown above represent the trial when individual mice first took Route 1 (first choice).Data are presented as the mean ± the standard error of the mean. (**b**) An example to define the trial when Route 1 retrieval occurs in a mouse. The trial of retrieval is shown by the red line. The moving average was calculated from the learning curve of Route 1. (**c**) Time changes in the total number of errors in Test 4 aligned to individual first choice (left) and time changes in the total number of errors in Test 4 aligned to individual Route 1 retrieval (right).

After the animals reached the learning criterion in the detour tasks, the two barriers were removed (Test 4) so that the condition of the maze was the same as in Test 1 (i.e., the optimal solution was again Route 1). Regardless of the previously preferred routes in the detour tests, all 14 animals successfully restored their most preferred route to Route 1 (Figs 1c and 9a). The animals required 9.3 ± 1.3 days (ranging from 3 days to 21 days; *n* = 14 animals) to reach the learning criterion. In 10 of 14 animals, the route ratio of Route 1 was zero on the first day (Fig. 9b), and the mean days elapsed until the animals first took Route 1 was 2.6 ± 0.5 days (Figs 9b and 10a). The maximum slope of the learning curve of Route 1 in Test 4 was 63 ± 3% per two days (Fig. 9c), which was larger than those of the most preferred routes in Test 1–3 (Test 1, 50 ± 3; Test 2, 42 ± 2; Test 3, 38 ± 4% per two days, all *P* < 0.05, Tukey’s test). The number of days to complete Test 4 was independent of the number of exploratory trials in the initial phase (Fig. 9d; *r* = 0.38, P = 0.18). The total number of VTE events did not significantly differ between the initial and the final phase (Fig. 9e; *t*_13_ = 0.86, P = 0.40). No correlation was found between the number of VTE events in the initial phase and the number of days in Test 4 (Fig. 9f; *r* = –0.028, P = 0.92).

In Test 4, there was a transient increase in erroneous choices during the middle phase of testing (Fig. 10a). The trial when each animal first encountered Route 1 was defined as the “first choice” (red dots in Fig. 10a), and the trial when each mouse showed the largest increase in the route ratio of Route 1 was defined as “Route 1 retrieval,” which was determined by the moving average method applied to the learning curve (Fig. 10b). The numbers of trial-and-error explorations were pooled from 14 mice and aligned relative to the trials of the first choice (Fig. 10c, left) and the Route 1 retrieval (Fig. 10c, right). The first choice-triggered change in the number of errors revealed that the number of errors increased by 256% immediately after the first choice compared to the baseline period of 60 trials before the first choice (Fig. 10c, left). The Route 1 retrieval-triggered change in the number of errors demonstrated that the increased errors after the first choice decreased by 49% after Route 1 retrieval (Fig. 10c, right). Taken together, pronounced erroneous behavior emerged specifically during the period from which the animals first took Route 1 to when they retrieved Route 1.

### Comparison of behavioral patterns across four tests

**Figure 11:**
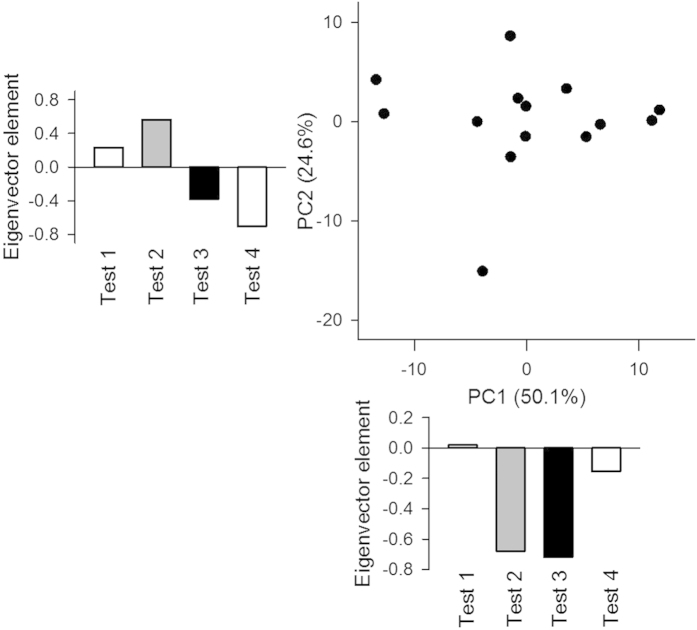
Comparison of testing days across four tests using PCA. In each mouse, the numbers of days in four tests (Fig. 1d) was defined as a four-dimensional vector and then decomposed into a two-dimensional vector. The first two PCs were plotted in a two-dimensional space. The bottom and left panels indicate the eigenvectors in the first and second PCs, respectively.

We compared the behavioral patterns across Tests 1–4. In each animal, the number of days spent in the four tests was regarded as a four-dimensional vector (Fig. 1d) and was subject to principal component (PC) analysis (Fig. 11). The first PC accounted for approximately half (50.1%) of the total variance, and its eigenvector elements representing Test 2 and Test 3 had negative values (–0.68 and –0.72, respectively). This result indicates a positive correlation between the number of days spent for Test 2 and Test 3; that is, animals that required more days for Test 2 also required more days for Test 3, independent of the days spent in Test 1 and Test 4. Plots in the first PC versus the second PC revealed no specific patterns among the animals.

**Figure 12:**
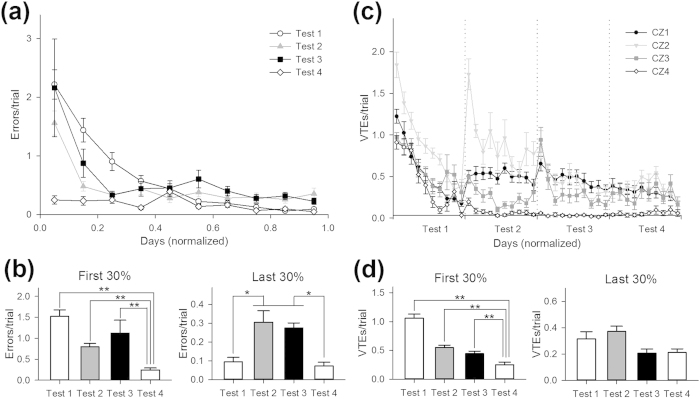
Erroneous behavior and VTE events across four tests. (**a**) The number of errors per trial. Days were normalized in each test ranging from 0 to 1. (**b**) The average number of errors in the initial (left) and last (right) phase of the four tests. *p < 0.05, **p *<* *0.01,* Steel-Dwass test. (**c**) The number of VTE events per trial in each choice zone (CZ), which corresponds to a graph combined from Figs 6a,7e,8e and 9e. (**d**) The average number of VTE events in the initial (left) and last (right) phase of the four tests. *p < 0.05, **p *<* *0.01,* Steel-Dwass test. All data are presented as the mean ± the standard error of the mean.

We next compared the number of errors across Tests 1–4 (Fig. 12a). In all of the tests, the number of errors in the initial phase (the first 30% of trials) was significantly higher than that in the final phase (the last 30% of trials) (Test 1, *t*_13_ = 9.0, *P* = 5.8 × 10^−7^; Test 2, *t*_13_ = 4.7, *P* = 4.3 × 10^−4^; Test 3, *t*_13_ = 2.8, P = 0.015; Test 4, *t*_13_ = 4.6, *P* = 4.8 × 10^−4^), confirming that the animals made more incorrect choices when they were first exposed to novel tasks. The number of errors in the initial phase in Test 4 was significantly lower than those in Test 1–3 (Fig. 12b, left; *P* < 0.01, Steel-Dwass test). This finding is because the mice were not yet aware that the barriers were removed and persisted on the previously established detour route. Interestingly, the numbers of errors in the last phase of Test 2 and Test 3 were significantly higher than those of Test 1 and Test 4 (Fig. 12b, right; *P* < 0.05, Steel-Dwass test), which indicates that errors were not fully suppressed in the barrier tasks compared to the no-barrier tasks. The errors that remained even after the animals settled on a near-best solution in Tests 2 and 3 may reflect the animal’s motivation to search for a better route.

In addition to the number of errors, we also compared the time course of VTE events across Tests 1–4 (Fig. 12c). The total number of VTE events in the initial phase monotonically declined as the tests progressed (Fig. 12d, left). This overall decrease suggests that the animals gradually became familiar with the context through repeated exposure to the maze. The total numbers of VTE events in the last phase in Test 2 were significantly higher than those in Test 3 and Test 4 (Fig. 12d, right; P < 0.05, Steel-Dwass test). Notably, VTE events in CZ1 in the last phase of Test 2–4 were higher than that of Test 1 (Test 1, 0.21 ± 0.03; Test 2, 0.46 ± 0.06; Test 3, 0.36 ± 0.07; Test 4, 0.23 ± 0.04), which is opposite of the overall decreasing trend of the total number of VTE events. This result has several implications: (i) the animals show VTE behaviors even at the choice point that is distant from the locus where the environment changes, which therefore can be regarded as predictive behaviors, and (ii) VTE behaviors become more pronounced once the animals have experienced environmental changes (Test 2–4). Thus, animals might become more suspicious to the present situation and take more deliberate decision-making strategies after facing situations in which their optimized strategies were interrupted.

**Figure 13:**
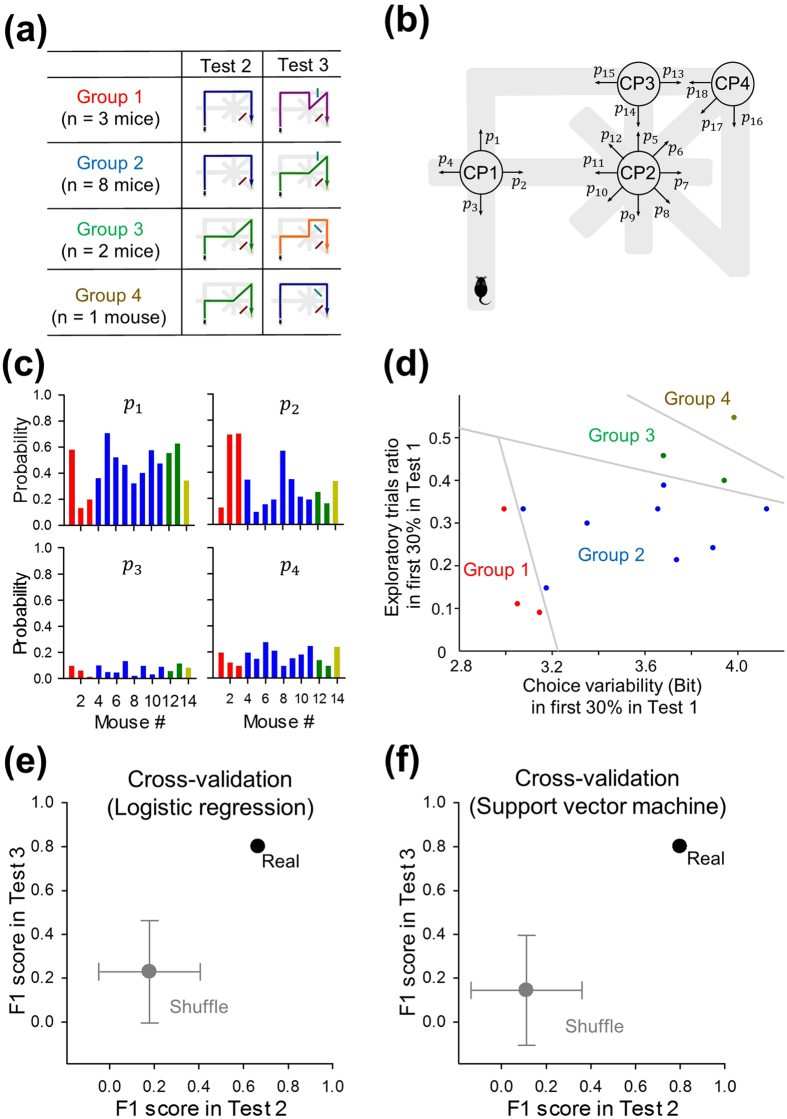
Early exploratory behaviors predict solution patterns. (**a**) Mice were classified into four groups depending their preferred routes in Tests 2 and 3. (**b**) For individual choice points (CP1-4), the choice probabilities *p*_*i*_ for all possible directions were measured using the datasets obtained from the initial phase (i.e., during the first 30% of the trials) of Test 1. (**c**) The choice probabilities at CP1 (*p*_1_, *p*_2_, *p*_3_, and *p*_4_) of all mice. Colors represent groups classified in a. (**d**) The numbers of exploratory trials in the initial phase of Test 1 were plotted against the choice variability *S*. Each dot indicates a single mouse. The four groups were completely separable using the linear support vector machine. (**e,f**) Leave-one-out cross-validation was performed using logistic regression (**e**) and the linear support vector machine (**f**). The predictability of the solutions arrived at by individual mice (F1 score) was significantly higher than for 1000 label-shuffled surrogates. Data are presented as the mean ± the standard deviation. For more details, see Methods.

Finally, we evaluated whether the early exploratory behaviors of the animals that had been initially exposed to the maze determined their choice strategies in subsequent detour tasks. As target (label) variables for prediction, all mice were classified into four groups based on their preferred routes in Tests 2 and 3 (Figs 1c and 13a). In each mouse, the probabilities that the mouse chose individual pathways at four choice points (*p*_1–18_) were calculated from datasets in the initial phase (i.e., during the first 30% of the trials) of Test 1 (Fig. 13b). For example, the probabilities *p*_1–4_ of all 14 mice are shown in Fig. 13c. The choice variability of each mouse was defined as the Shannon entropy of *p*_1–18_ (see Methods). It takes a higher value when the mouse explored more evenly and a lower value when it repeated the same exploratory pattern more frequently. Interestingly, the four groups were completely separable by three straight lines in the two-dimensional space of exploratory trial number during the initial phase of Test 1 *versus* the choice variability (Fig. 13d). The separation lines were determined using the linear support vector machine. This separation is significant because the probability that three straight lines can completely separate four groups when mouse IDs are randomly shuffled is as low as 0.013. Therefore, we examined whether the preferred routes of individual mice in Tests 2 and 3 could be predicted based on their choice variabilities and the number of exploratory trials in the initial phase of Test 1. To this end, we employed leave-one-out cross-validation analyses with two supervised machine learning algorithms: logistic regression (Fig. 13e) and a linear support vector machine (Fig. 13f). We adopted five variables: the choice variabilities at four individual choice points and the number of exploratory trials in the initial phase of Test 1. Prediction performance was evaluated using the F1 score and was compared to the chance level, which was estimated from 1000 label-shuffled surrogates (for details, see Methods). In both Tests 2 and 3, the F1 scores were significantly higher than the chance level (logistic regression: *P* = 0.063 in Test 2 and 0.043 in Tests 3; linear support vector machine: *P* = 0.059 in Test 2 and 0.035 in Tests 3). These results suggest that the early exploration frequency and the early choice variability in Test 1 determine the performance of choice behavior after mice are forced to change navigational strategy. Specifically, given than groups 3 and 4 settled on shorter routes than groups 1 and 2, mice that had explored the maze more heavily and extensively in the early phase were more likely to establish better routes.

## Discussion

In this study, we analyzed the behavioral patterns of mice in a series of maze problems. Compared with tasks used in previous studies, such as T-maze tasks, a zigzag maze task^14^, and Tolman’s detour tasks^5^,^15^, our maze contained more complicated factors, including an asymmetrical structure, multiple correct routes, heterogeneous choice points, dead ends, and blocking barriers, which demands mice to make various decision-making steps depending on the condition of the maze. All 14 mice completed the four tests, indicating that mice have the ability to find appropriate solutions even under a high degree of complexity.

In Test 1, all of the mice eventually learned to take the same optimized route, but the days spent reaching the learning criterion varied among mice, ranging from 3 to 18 days. Remarkably, these durations were negatively correlated with the frequency of exploratory trials observed in the initial phase of Test 1. Likewise, the frequency of exploratory behavior in the initial phase of the detour task (Test 2) was predictive of later task performance. These results suggest that trial-and-error searching is not a wasteful action but may facilitate correct decision making in the later period. It is worth noting that the solutions on which mice eventually settled in the detour tasks differed across individual mice. This variation suggests that once mice established their own near-best strategy, they persisted in the selected behavioral pattern and rarely took the other routes. Importantly, many mice preferred Route 4, a less-choice-point, peripheral route, whereas they rarely chose shorter routes, such as Route 2 and Route 3. This preference suggests that travel distance is not the sole determinant of behavioral patterns, but rather that mice tend to take a strategy that requires less deliberation and fewer choice costs.

It has been widely assumed that spatial mazes are solved by a cognitive map, which internally represents the associational relevance of environmental cues^1^,^2^. Our study provides several insights into the cognitive map theory. First, we observed a sign of latent learning, a learning process without the emergence of apparent purposeful actions^7^. Even on the first day of Test 1, the mice were already skilled and were able to take efficient routes. This ability could be achieved by recalling the internal memory of the spatial organization of the maze that they had learned in the habituation training. Second, we observed VTE behaviors while the animals were committed to a decision at a choice point. These vicarious behaviors presumably reflect hesitation, conflict, and deliberation of future consequences and therefore are considered to temporally hold information online and benefit decision-making by predicting outcomes before taking an action^16^,^17^. In accordance with the literature, VTE events occurred most frequently in the initial testing phases and gradually decreased as learning proceeded^9^,^13^. Compared to trial-and-error exploratory behaviors, the frequency of VTE events correlated less with task performance. These results suggest that trial-and-error searching-behaviors, rather than VTE behaviors, may contribute to learning an efficient route.

After a familiar route was suddenly blocked, mice could flexibly shift to another appropriate route in the first trial. From the beginning of the detour conditions, individual trajectories were not random, but could be classified into seven unicursal routes. These results suggest that animals have a sense of accurate orientation toward the reward position and thereby can design a novel alternative route in an efficient manner. This ability implies the existence of an internal model of the environment for deliberating expectancies associated with their choices. When the blocked routes became available, exploratory behaviors increased immediately after the mice first restored the optimized route. The transient exploration mode may represent information-seeking behaviors to scrutinize the efficiency of each alternative option before determining the final strategy.

In conclusion, our study demonstrated that early trial-and-error exploration is not merely a noisy action, but it serves as a crucial behavior that improves future problem solving in mouse spatial mazes. The results support the idea that slow learners in the training sessions performed better in finding alternative strategies in the subsequent test sessions. These salient behavioral features are potentially associated with cognitive learning processes and purposeful behaviors. We suggest their coordinated integrations may underlie animal’s ability to flexibly choose strategies that meet the instantaneous demands of the current situations.

## Methods

### Animals

A total of 16 naïve C57Bl/6J male mice (7–16-week-old; SLC, Shizuoka, Japan) weighing 21–26 g were housed one per cage and maintained on a 12-h light/12-h dark schedule with lights off at 6:40 PM. They had free access to water and were food-deprived to 90% of ad libitum body weight. All experiments were performed with the approval of the animal experiment ethics committee at the University of Tokyo (approval number 24–10) and according to the University of Tokyo guidelines for the care and use of laboratory animals. Of the total 16 animals used, 1 animal was tested inappropriately and 1 animal did not complete the tests successfully as it did not meet the learning criteria as described below. These two animals were thus excluded from further analyses.

### Apparatus

The maze apparatus used in this study is shown in Fig. 1a. The maze was made of ABS resin, and all of the alleyways were 6 cm long with a transverse wall (15 cm in height). The first path initiated from the start position, branched off to two alleys in the middle of the path; the left alley was a dead end, and the right alley was connected to an 8-arm radial maze. The first path ended by turning right, forming the outer peripheral path. The radial maze consisted of a central circle (26 cm diameter) and 8 alleys (23 × 6 cm) radiating symmetrically outward, with three alleys connecting to three different portions of the middle of the outer peripheral path, and another alley directly led to the goal position where a food pellet reward was placed in behavioral tests. The four other alleys terminated at a dead end. In some of the behavioral tests, some paths were blocked by introducing barriers (10 cm in width; 15 cm in height).

### Habituation to the maze

From the first day of food restriction, a mouse was daily placed on the start position in the maze without adding any barriers and allowed to freely forage for 15–20 food pellets (20 mg) that were placed at individual nodes of the maze for 10 min (Supplementary Fig. 1a). The initial habituation was repeated for 7–8 days.

### Behavioral tests

In all of the following behavioral tests, a food pellet reward (20 mg) was placed only at the goal location for every trial. The mouse was subjected to 20 trials per day. In Test 1, each trial started by placing the mouse at the start position without adding any barriers in the maze. A trial ended when the animal consumed the food reward or explored in the maze without reaching reward location for more than 60 s. The animal was then put back into a homecage and the next trial was initiated after an inter-trial interval of 1–2 min. In the maze task, there were 7 possible unicursal routes that the animal could take without visiting the same nodes more than twice (Fig. 1b). The shortest route from the start to the goal was Route 1, and all 14 animals eventually took this route in most of the trials (Fig. 1c). Test 1 was repeated every day until the animal took Route 1 in 15 trials per day (75% of the total trials) for 2 consecutive days. After completing Test 1, Test 2 was initiated with a barrier placed at segment K (1^st^ Barrier). Except for the addition of the barrier, all of the behavioral procedures were similar to those in Test 1. In Test 2, the most chosen detour route was defined on each day. Tests were repeated every day until the animal took the same detour route in 15 trials per day for 2 consecutive days. The most chosen detour route was defined as the most preferred route. If the animal could not achieve the criterion within 21 days, Test 2 was completed and the route most chosen by the animal at the 21^st^ day was defined as the most preferred route. Eventually, 11 and 3 animals showed their most preferred route as Route 4 (a route consisting of the full outer peripheral path) and Route 2 (a route including an alley of the radial 8-arm maze and the last part of the outer peripheral path), respectively (Fig. 1c). After completing Test 2, Test 3 was performed by adding another barrier (2^nd^ Barrier). The position of the 2^nd^ Barrier was determined according to the most preferred route that the animal took in Test 2 (i.e., a barrier was introduced at segment J or segment L in the case that the most preferred route in Test 2 was Route 4 or Route 2, respectively). Except for the existence of the two barriers, all of the behavioral procedures were similar to those in Test 2. In Test 3, as in Test 2, the most chosen detour route was defined on each day, and the tests were repeated every day until the animal took the same most chosen detour route in 15 trials per day for 2 consecutive days. The most chosen detour route was defined as the most preferred route. If the animal could not achieve the criterion within 21 days, Test 3 was completed and the route most chosen by the animal at the 21^st^ day was defined as the most preferred route. In Test 3, the most preferred detour routes varied depending on the animals and the barrier positions, including Routes 2, 4, 5 and 7. After completing Test 3, Test 4 was initiated by removing the two barriers, meaning that Test 4 was similar to Test 1. In Test 4, all 14 animals eventually showed their most preferred route corresponded with the shortest route (Route 1), similar to Test 1. Test 4 was repeated every day until the animal took Route 1 in 15 trials per day for 2 consecutive days. After all 20 trials, the maze was cleaned with ethanol; that is, cleaning was performed once per day per animal, unless otherwise specified. In experiments shown in Supplementary Fig. 3, the maze was cleaned every trial. Animals’ trajectories were tracked at 25 Hz using a video camera located on the ceiling above the experimental area.

### Analysis of animal trajectory

Representative trajectories observed from a mouse are shown in Fig. 2. To analyze the trajectories, the maze was divided into multiple segments (edges), and individual segments were labeled with alphabet characters A–O (Fig. 3a). Each trajectory in a trial was described as a character string, termed as a target string (Fig. 3c). Each of the seven unicursal routes shown in Fig. 1b was defined as a template and converted into a character string in the same manner, termed a template string (Fig. 3b). For a target string and the trajectory in a trial, we analyzed the numbers of mismatches between the target string and individual template strings (examples are shown in Fig. 3c). A route with the template string having the least mismatch was defined as the closest route, and the number of the least mismatch was defined as the number of errors. If the number of errors was less than 1, the animal’s trajectory was defined as the closest route. If the number of errors was more than 2, the trajectory was not classified into any routes and the trial was considered “an exploratory trial.” On each day, the probability of choosing a route was defined as a route ratio (Fig. 4a).

### Detection of vicarious trial-and-error (VTE) behavior

Vicarious trial-and-error (VTE) behavior was measured as previously described^18^. We focused on four zones that were extended 7.5 cm on all sides from a hinge region including a choice zone, labeled CZ1–4 (Fig. 5b). The animal’s velocity in the *x* and *y* coordinates was calculated using the adaptive windowing algorithm (Fig. 5a)^19^, and the corresponding angular velocity *Phi* was defined as the arctangent of these two components (Fig. 5b). From a series of *Phi*, the angular acceleration *dPhi* was calculated using the adaptive windowing algorithm^19^. In each trajectory, an integral of the absolute values of *dPhi* (i.e., *IdPhi*) was calculated throughout the period while animals traveled in a choice zone (Fig. 5c, left). Integrated angular velocity (IAV) was defined as log(*IdPhi*). In each choice zone and each travel angle, a distribution of IAV ranging from the lowest value to the mode value was fitted by a Gaussian curve (Fig. 5c, right). A VTE event was defined if a trajectory in a choice zone had an IAV value higher than the 5% significance level that was estimated from the fit to the distribution to which the trajectory belonged.

### Moving average

In Test 4, a trial of Route 1 retrieval was estimated using the moving average method as previously described^20^. The probability of a Route 1 choice at trial *k* was calculated as follows:


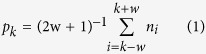


where 2 *w* + 1 is the length of a series of overlapping windows (here, *w* = 4) and *n*_k_ corresponds to 1 and 0 when the animal took Route 1 or other routes at trial *k*, respectively. The trial with Route 1 retrieval was defined as the middle trial in the windows for which the probability of determining the observed number of correct responses was below 0.05, assuming that the chance probability of Route 1 choice is 0.125 (1/8).

### Choice variability

In each mouse, a probability for each alleyway at a choice point in a hinge region (CP_1–4_, see Fig. 13b) was calculated from the dataset obtained in the first 30% of Test 1 trials (for the numbering of probability *p*, see Fig. 13b,c). No conditional probability was taken into account. Based on the set of all choice probabilities *p*_*1–18*_ for each mouse, a weighted Shannon entropy *S* was calculated and defined as a “Choice variability” as follows:


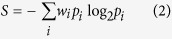


where 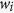 is 1 if the choice direction of *p*_*i*_ corresponds any forward directions along the seven unicursal routes (i.e., 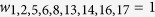). In contrast, 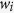 is 0 if the selected alley of *p*_*i*_ is a dead end or the direction of *p*_*i*_ never matches any forward directions in the seven unicoursal routes (i.e., 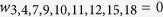).

### Logistic regression

At each choice point, a choice variability x_j_ was calculated from a series of choice probabilities *p*_*i*_ arising from the corresponding choice point CP_*j*_.


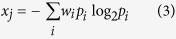


In a mouse_*k*_, a feature vector **x**^**(k)**^ was defined as follows:


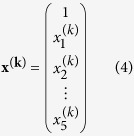


where 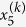 is the number of exploratory trials observed in the initial phase (i.e., during the first 30% of the trials) of Test 1.

L1 regularized logistic regression was applied to predict mouse types that were classified by their optimized routes, as observed in Tests 2 and 3 (see Fig. 13a). In this analysis, the vector *x* was used as a training dataset and a label data 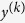 was set to 1 or 0 depending on whether the optimized route of mouse_*k*_ in Test 2 was Route 2 or Route 4, respectively. In Test 3, 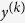 was set to be 1 or 0 depending on whether the optimized route was Route 2 or Route 7, respectively. Using a logistic regression parameter vector 
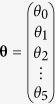
, the cost function *J(***θ**) was calculated as follows:


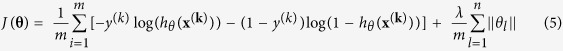


where *m* = 14 (the number of mice) and *n* = 5 (the number of feature variables used in a vector). A sigmoid function 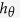
was defined as follows:


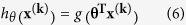



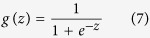


The regularization parameter 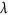 was searched using F1 score (see Cross-validation) and determined to be 0.0042424. To optimize the parameter **θ** to minimize the cost function *J*(*θ*), a partial derivative was calculated as follows:


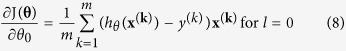



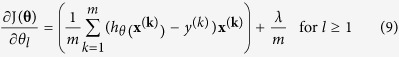


The optimization was performed using the Octave function *fminunc* (http://www.gnu.org/software/octave/).

### Linear support vector machine (SVM)

The same dataset used in logistic regression analysis was analyzed using a linear support vector machine (SVM). The Octave SVM-code is available at LIBSVM (http://www.csie.ntu.edu.tw/~cjlin/libsvm/21. SVM-type was set as C-“Support Vector Classification” (C-SVC), Kernel type was set as “linear”, and Parameter C was searched and set to be 35.

### Cross-validation

Leave-one-out cross-validation was used to predict the optimized routes in Tests 2 and 3. Analyses were performed independently for each test period. For all 14 mice, the classifier learned and predicted whether the optimized route was Route 2 or Route 4 in Test 2. For the 11 mice that chose Route 4 as the optimized route in Test 2 (classified as Type 1 and Type 2 in Fig. 13a), the classifier learned and predicted whether the optimized route would be Route 2 or Route 7 in Test 3. F1 scores were calculated to evaluate the accuracy of the prediction and were compared with those obtained from 1000 label-shuffled surrogates.

### Statistical Analysis

All data are presented as the mean ± the standard error of the mean (SEM), unless otherwise specified.

## Additional Information

**How to cite this article**: Igata, H. *et al*. Early Failures Benefit Subsequent Task Performance. *Sci. Rep.* **6**, 21293; doi: 10.1038/srep21293 (2016).

## Supplementary Material

Supplementary Information

Supplementary Information
