# Supplementary material for: Early Failures Benefit Subsequent Task Performance
Source: Sci Rep. 2016 Feb 17;6:21293. doi: 10.1038/srep21293 (PMC4756702; doi:10.1038/srep21293)
Supplement: Supplementary Information [file srep21293-s2.doc]

Supplementary information

**Early Failures Benefit Subsequent Task Performance**

Hideyoshi Igata, Takuya Sasaki, Yuji Ikegaya

This file includes 3 supplementary figures.

**
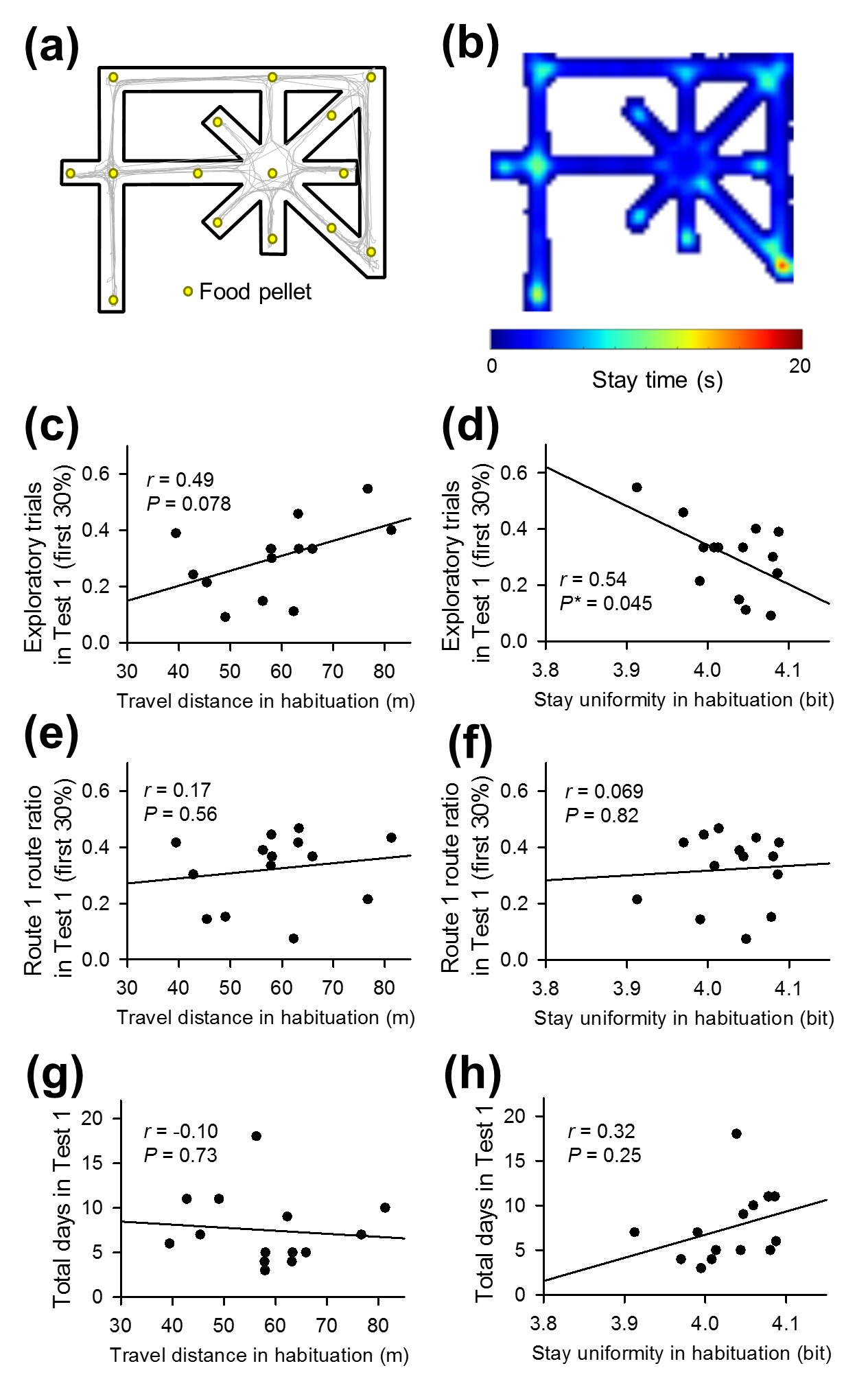
**

**Supplementary Figure S1.** Behavioral performance during 7-day habituation periods. (a) A representative trajectory of an animal in a 10-min habituation session. In habituation sessions, food pellets were placed at all nodes as shown in yellow dots, and animals were allowed to freely forage throughout the maze for 10 min. (b) The probability density of sojourn time in the maze averaged across all animals. In each animal, “stay uniformity” throughout the maze was defined as the entropy:
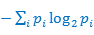
, where
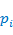
 is the probability density of segment *i* (see Figure 3a for the definition of 15 segments and 5 hinge segments). The stay uniformity takes 0 bit if an animal remains at one segment throughout the recording and 4.3 bit if an animal completely evenly covers all segments. The stay uniformity of the 15 animals ranged from 3.9 to 4.1 with an average of 4.0, suggesting that all animals showed homogeneous probability distribution. (c, d) The number of exploratory trials in the first 30% period in Test 1 tend to correlate with travel distance (c) and stay uniformity (d) during habituation periods. Each dot represents a mouse. (e, f) No correlations were found between the route ratio of Route 1 in the first 30% period in Test 1 and travel distance (e) and stay uniformity (f) during habituation periods. (g, h). No correlations were found between total days to reach the learning criterion in Test 1 and travel distance (g) or stay uniformity (h) during habituation periods.

**
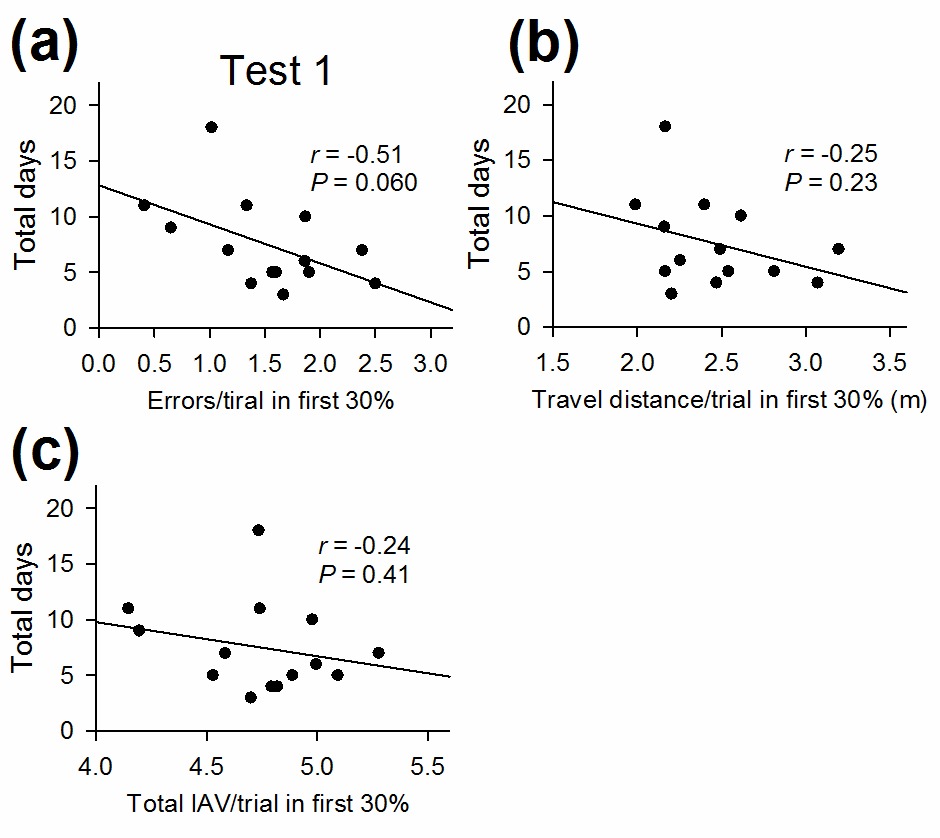
**

**Supplementary Figure S2.** Learning time course and behavioral characteristics in Test 1. Data were shown same as in Figure 4d, but days to reach the learning criterion were plotted for the number of errors (a), the travel distance (b), and the total IAV (integrated angular velocity; for detail, see Figure 5) (c) per trial in the first 30% periods in Test 1.


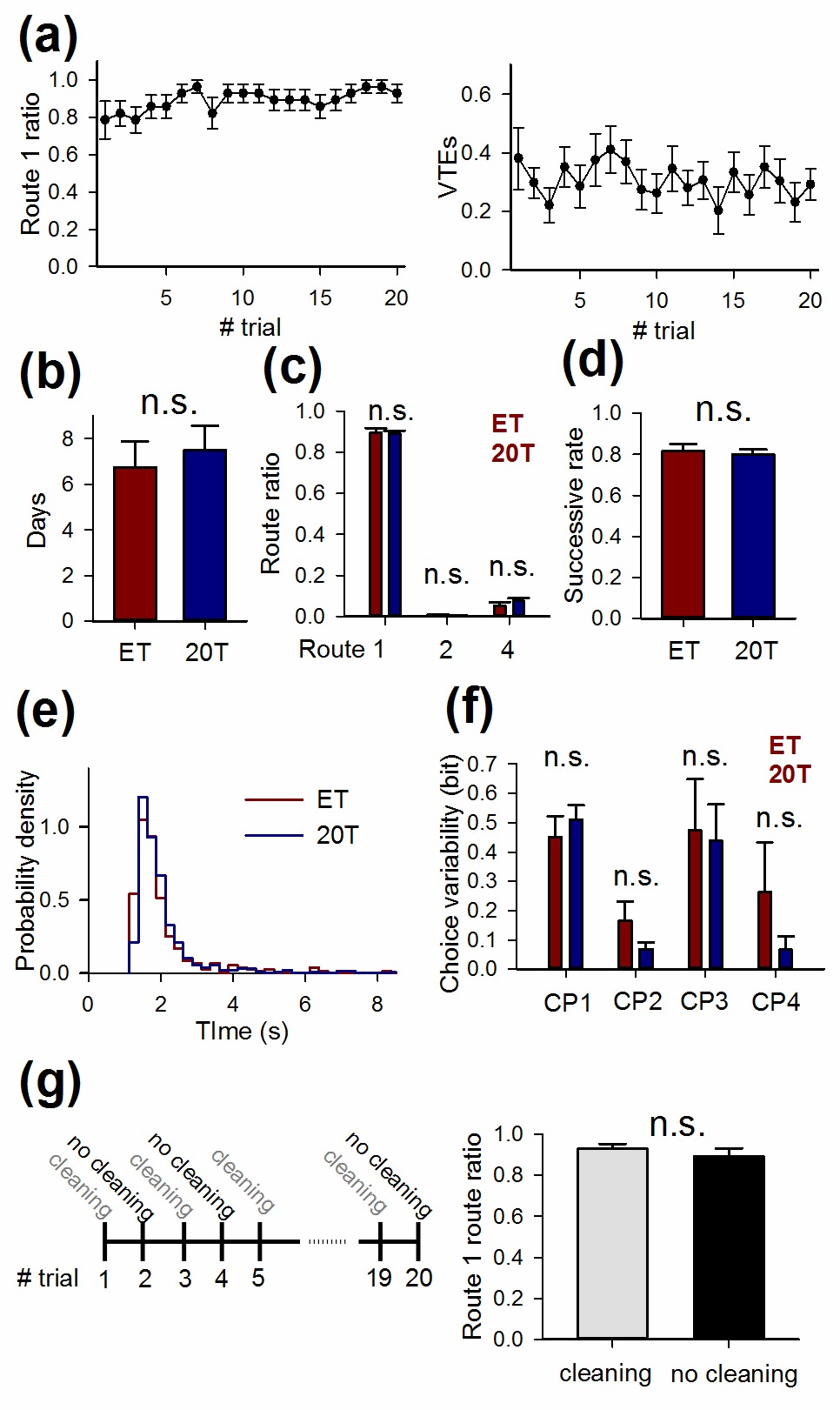


**Supplementary Figure S3.** Behavioral performance and motor activity are not affected by the frequency of cleaning the maze. (a) Time changes in the average route ratio of Route 1 (left) and the average number of VTE events (right) in the last two days of Test 1. One-way ANOVA revealed that these parameters do not vary across trials (Route 1 route ratio, *F*(19,279)=0.91, *P* = 0.57; VTE events, *F*(19,279)=0.63, *P* = 0.88). (b) The number of days to reach the learning criterion in animal groups tested in the condition in which the maze was cleaned every trial (*n* = 8 animals, ET group) and in the condition in which the maze was cleaned after completing all 20 trials (*n* = 14 animals, 20T group) (t_20_ = 0.45, *P* = 0.66). (c) The percentage of taking Route 1, Route 2, and Route 4 in the last two days (Route 1, t_20_ = -0.23, *P* = 0.82; Route 2, t_20_ = -0.60, *P* = 0.55; Route 4, t_20_ = 0.92, *P* = 0.37). (d) The percentage of taking a same route in two successive trials (t_20_ = -0.39, *P* = 0.70). (e) The distribution of the duration for one trial in the last two days (*D*_max_ = 0.098, *P* = 0.058, Kolmogorov-Smirnov test). (f) The choice variability at individual choice points in last two days (CP1, t_20_ = 0.62, *P* = 0.54; CP2, t_20_ = -2.07, *P* = 0.052; CP3, t_20_ = -0.45, *P* = 0.65; CP4, t_20_ = -1.42, *P* = 0.16). (g) The maze was cleaned every 2 trials. Route 1 route ratio was not significantly different between trials after cleaning (cleaning trials) and trials without cleaning (no cleaning trials) (t_6_ = -1.37, *P* = 0.22).

**Supplementary Movie 1.** Three typical trials in the last two days of Test 1. The first and the second trials are recorded from the same animals at the initial phase and the last phase in the same day without cleaning the maze. The third trial shows behavior after cleaning the maze (corresponding with Supplementary Figure 3). Time counting starts at the first choice point. The movie shows the mice run through the maze quickly at the nearly maximal speed without showing behavioral signs related to explorations of scent cues.
